# Overexpression of the circadian gene Bmal1 regulates the Nrf2/HO-1 oxidative stress pathway to alleviate inflammation and apoptosis in PC12 cells following cerebral ischemia-reperfusion injury

**DOI:** 10.1097/MD.0000000000042763

**Published:** 2025-06-13

**Authors:** Fukang Zeng, MengJuan Wang, Zhong Li, Yuxing Zhang

**Affiliations:** a Xiangxi Tujia and Miao Autonomous Prefecture Ethnic Chinese Medicine Hospital, Jishou, Hunan, China; b Department of Neurology, The First Hospital of Hunan University of Chinese Medicine, Changsha, Hunan, China; c Hunan University of Chinese Medicine, Changsha, Hunan, China; d Department of Neurology, The University of Texas Health Science Center at Houston, Houston, TX.

**Keywords:** Bmal1 gene, cerebral ischemia-reperfusion injury, oxidative stress

## Abstract

The circadian clock gene brain and muscle Arnt-like 1 (Bmal1) plays a crucial role in cerebral ischemia-reperfusion injury. Therefore, we established stable transfections of Rat adrenal pheochromocytoma cells (PC12) to overexpress the Bmal1 gene and a negative control using lentivirus. An in vitro model of cerebral ischemia-reperfusion injury was created through oxygen-glucose deprivation/reoxygenation (OGD/R) induction. The cells were divided into 4 groups: control, OGD/R, OGD/R with Bmal1 negative expression, and OGD/R with Bmal1 overexpression. The mRNA expression level of Bmal1 was measured using quantitative reverse transcription polymerase chain reaction. Protein levels of Bmal1, Nuclear factor erythroid 2-related factor 2 (Nrf2), heme oxygenase-1 (HO-1), BCL2-Associated X, B-cell lymphoma-2 (Bcl-2), and cysteine-dependent aspartate-directed protease 3 were assessed via Western blotting. The levels of inflammatory cytokines interleukin-6, and interleukin-1β in the cell supernatant were quantified using ELISA. Apoptosis rates were analyzed by flow cytometry, and intracellular reactive oxygen species levels were measured using a fluorescent probe. Following OGD/R induction, Bmal1 gene and protein expression levels were reduced in PC12 cells. After lentiviral transfection, mRNA and protein expression levels of Bmal1 significantly increased in the overexpression model. Bmal1 overexpression down-regulated apoptotic proteins BCL2-Associated X and cysteine-dependent aspartate-directed protease 3, up-regulated the antiapoptotic protein Bcl-2, reduced apoptosis, and inhibited the release of inflammatory factors interleukin-6 and interleukin-1β following OGD/R. Further experiments indicated that Bmal1 overexpression activated proteins in the Nrf2/HO-1 oxidative stress signaling pathway, reducing intracellular reactive oxygen species release. This study demonstrated that Bmal1 overexpression inhibits inflammatory responses and apoptosis in PC12 cells after ischemia/reperfusion injury by regulating the Nrf2/HO-1 oxidative stress signaling pathway.

## 1. Introduction

Ischemic stroke is a common condition where disrupted cerebral vascular blood supply causes ischemia, hypoxia, and neurological deficit, making it a leading cause of global death and disability.^[1–3]^ The primary goal in treating acute cerebral infarction is timely restoration of reperfusion in the ischemic area; however, rapid reperfusion can worsen brain function damage, leading to cerebral ischemia-reperfusion injury (CIRI).^[4]^ The complex pathophysiological mechanism of CIRI are primarily associated with mitochondrial energy metabolism disorder, excitatory amino acid toxicity, ion imbalance, oxidative stress, inflammation, apoptosis, and blood–brain barrier damage. Ischemia and hypoxia in brain tissue during reperfusion rapidly generate free radicals, triggering the release of inflammatory factors, intracellular calcium overload, and the activation of apoptotic genes, ultimately causing neuronal apoptosis.^[5]^ Ischemic stroke populations impose a significant economic burden on countries, particularly low- and middle-income nations, while high-income countries, due to their higher socioeconomic status and greater investment in healthcare, face relatively lower economic burdens. To address ischemic stroke and CIRI in underserved populations across low-, middle-, and high-income countries, coordinated efforts are needed on multiple fronts. First, strengthen primary prevention to reduce incidence rates and optimize acute-phase treatment to lower mortality and disability rates. Second, adjust policies to prioritize resource allocation toward low- and middle-income countries, bridging gaps in care. Finally, increase investment in poststroke rehabilitation to improve patients’ quality of life. Countries with varying income levels should tailor strategies to local conditions, integrating technology promotion, cost control, and international collaboration to progressively achieve equitable and effective stroke prevention and treatment.^[6]^

Oxidative stress (OS) plays a key regulatory role in the pathology of CIRI,^[7]^ OS refers to the imbalance between oxidative and antioxidative states within cells, caused by excessive reactive oxygen species (ROS) or impaired antioxidant defenses, leading to cellular damage. During cerebral ischemia and reperfusion, blood flow restoration rapidly activates vascular endothelial and neuronal cells, generating large amounts of ROS and inducing OS. OS directly damages lipids, proteins, and DNA, while also promoting inflammation and apoptosis via multiple signaling pathways, exacerbating brain damage. OS directly damages cellular components like lipids, proteins, and DNA, and activates inflammation and apoptosis, worsening brain damage.^[8,9]^ Nuclear factor erythroid 2-related factor 2 (Nrf2) is a crucial transcription factor that mitigates OS damage. Upon OS stimulation, Nrf2 is released from Kelch-like ECH-associated protein 1 and translocates to the nucleus, where it binds to the antioxidant response element and activates the transcription of antioxidant enzymes like heme oxygenase-1 (HO-1) and NAD(P)H Dehydrogenase [quinone] 1, which scavenge excess ROS and reduce oxidative damage. These antioxidant enzymes scavenge excess ROS in cells, reducing OS.^[10]^ HO-1, a key gene regulated by Nrf2, plays a crucial role in protecting cells from oxidative stress, inflammation, and apoptosis.^[11,12]^

The circadian clock, or circadian rhythm, is an intrinsic mechanism that regulates the physiological rhythms of living organisms.^[13]^ Regulated by complex circadian clock genes and a network of clock-controlled genes, the circadian clock generates rhythmic oscillations, maintains homeostasis, and influences physiological and pathological processes.^[14]^ Bmal1, a core clock gene, plays a key role in this regulation.^[15]^ Disturbances in Bmal1 gene expression during ischemia-reperfusion injury disrupt the OS response, exacerbating cellular damage.^[16,17]^ The Bmal1 gene may serve as a crucial target for regulating OS and providing protection against ischemia-reperfusion injury and other diseases. However, the specific role of Bmal1 in CIRI and its underlying mechanisms remain unclear. Pheochromocytoma cells (PC12), derived from rat adrenal medulla pheochromocytoma, are widely used in in vitro studies of neurological diseases.^[18]^ In this study, we established a Bmal1 gene overexpression model in PC12 cells through lentiviral transfection, along with a negative control (NC) model. Additionally, we induced an in vitro model of CIRI using oxygen-glucose deprivation/reoxygenation (OGD/R) to investigate the protective mechanism of Bmal1. This study aims to investigate the protective mechanism of the Bmal1 gene in CIRI. This study provides experimental evidence and theoretical support for the application of Bmal1 gene regulation strategies in clinical treatment, potentially advancing clinical translational research in this field.

## 2. Materials and methods

### 2.1. Cell origin and culture

PC12 cells used in this study were purchased from the Chinese Academy of Science Cell Bank (TCR9, China). The cells were cultured in Dulbecco’s Modified Eagle Medium supplemented with 10% fetal bovine serum and 1% penicillin and streptomycin, and incubated at 37°C in a 5% CO_2_ and 95% O_2_ atmosphere. The PC12 cells were subcultured, and the culture medium was refreshed every 48 to 72 hours. Finally, the cells used for the subsequent experiments were in the logarithmic growth phase.

### 2.2. Cell OGD/R model

Before oxygen-glucose deprivation treatment, the culture medium was replaced with glucose-free DMEM and incubated in a 5% CO_2_ and 95% N_2_ atmosphere at 37°C for 4 hours. Subsequently, the cells were returned to a normal medium containing glucose and incubated in an atmosphere with O_2_ for 24 hours of reoxygenation,^[19]^ cell morphology was examined using the inverted microscope (ZEISS, Germany).

### 2.3. Bmal1 overexpression lentivirus vector infection

The lentiviral vectors for Bmal1 overexpression and NC were constructed by Hanbio Biotechnology Co., Ltd. (China). All procedures were conducted following the manufacturer’s instructions. In summary, PC12 cells were prepared as a suspension and inoculated into a 6-well plate at a density of 6–8 × 10^4^ cells per well for 24 to 36 hours. Once the cells reached a density of 40% to 50%, an appropriate volume of the lentivirus infection reagent was added to the culture medium according to MOI = 30. After 24 hours, the medium was replaced with a complete medium. Transfection efficiency was assessed 72 hours post-transfection by measuring the expression of green fluorescent protein. Subsequently, the PC12 cells were subcultured in a medium containing 2 ng/mL puromycin for 7 days, achieving a transfection efficiency exceeding 95%. Untreated PC12 cells served as the blank control groups. The transfection efficiency was then evaluated using Western blotting and real-time quantitative polymerase chain reaction.

### 2.4. Western blotting

Cell proteins from each group were extracted following the instructions of the Enhanced Ripa cracking liquid (ApplyGEN, China), which contains PMSF (CWBIO, China). Protein concentration was measured using a BCA protein Quantitation Kit (Elabscience, China). After adding the loading buffer (CWBIO, China), the mixtures were denatured at 100°C for 20 minutes. A 10 μL of protein were loaded per lane separated using a 10% sodium dodecyl sulfate-polyacrylamide gel, then transferred to a 0.45 mm PVDF membrane (Millipore, China). The membrane was blocked with a 5% skimmed milk solution for 1 hour at room temperature and then incubated overnight at 4°C with specific antibodies. The primary antibodies used were anti-Bmal1 (Abcam, 1:2000), anti-Nrf2 (Proteintech, 1:5000), anti- HO-1 (Proteintech, 1:5000), anti-cysteine-dependent aspartate-directed protease 3 (anti-Caspase-3) (Proteintech, 1:2000), anti-Bcl-2 (Proteintech, 1:3000), BCL2-Associated X (Bax; Proteintech, 1:10000), and anti-β-actin (Proteintech, 1:8000). On the second day, the membrane was incubated with anti-rabbit horseradish peroxidase- (HRP-) conjugated secondary antibodies (Elabscience, China) for an additional 1.5 hours at room temperature. Signal detection was performed using the Efficient Chemiluminescence Kit (GEN-VIEW, China), and results were recorded with the Bio-Rad ChemiDoc MP Gel Imaging System. The band density of specific proteins was quantified after normalization to the density of β-actin.

### 2.5. Real-time quantitative polymerase chain reaction

Total RNA was extracted from each group using the Ultra-Pure Total RNA Extraction Kit (SimGen,China). Reverse transcription was performed using a reverse transcription kit (Novoprotein,China). Quantitative reverse transcription polymerase chain reaction was conducted using the CFX96Touch (Bio-Rad, USA) following the manufacturer’s protocol. The mRNA levels of each target gene were normalized to β-actin. The primers used are provided in Table 1. The reaction conditions included 40 cycles at 95°C for 90 seconds, 95°C for 10 seconds, and 60°C for 30 seconds. The relative expression levels of the desired gene was analyzed using the 2^−ΔΔCt^ method.

**Table 1:** Sequences of the primers for RT-qPCR.

Name	Forward primer sequence(5'-3')	Reverse primer sequence(5'-3')
Bmal1	AGGCCTTCATTGCACCTTCC	ATTTTGTCCCGACGCCTCTT
β-Actin	CACCCGCGAGTACAACCTTC	CCCATACCCACCATCACACC

Bmal1 = brain and muscle Arnt-like 1, RT-qPCR = quantitative reverse transcription polymerase chain reaction.

### 2.6. ELISA assays

The levels of interleukin-1β (IL-1β), interleukin-6 (IL-6; CSB-E04640r, CSB-E08055r, Cusabio, China) in the supernatants of each cell group were measured using ELISA. The optical density values of the samples at 450 nm were determined using the standard curve. The concentrations of the inflammatory factors in each sample were determined using a standard curve.

### 2.7. Flow cytometry

PC12 cells groups were collected, and the concentration was adjusted to 3 × 10^5^ cells/ml using PBS. Subsequently, 200 μL of AnnexinV-APC staining solution (C1062, Beyotime, China) was added to 1 mL of the cell suspension, followed by the addition and mixing of 20 μL of propidium iodide staining solution. Cells were analyzed using flow cytometry (CytoFLEX AW39253, Beckman, Brea) and counted according to the following formula, apoptotic index apoptotic cells = number/total number of cells detected × 100%.

### 2.8. Fluorescent probe to detect ROS content

For each group, a diluted solution of DCFH-DA (10 μmol/L) from the ROS Assay Kit (CA1410, Solarbio, China) was added. The cells were incubated for 30 minutes in the dark to ensure adequate contact between the DCFH-DA probe and the cells. After aspirating and discarding the dye, the cells were washed 3 times with PBS. Subsequently, DAPI was added for nuclear staining. The cells were then observed and imaged using a fluorescence microscope. Finally, the fluorescence intensity of the cells was quantified using ImageJ software.

### 2.9. Statistical analysis

Statistical analysis and graphical representations were conducted using GraphPad Prism 8.0.2. Data are presented as the mean ± Standard Deviation from at least 3 independent experiments. we applied a paired *t* test to result 3.1, used 1-way ANOVA for data meeting normality and homogeneity of variance in results 3.2 to 3.4, and employed the Kruskal-Wallis *H* test for data violating these assumptions, with Tukey HSD tests for post hoc verification. *P*-value < .05 indicated that the difference was statistically significant.

## 3. Results

### 3.1. Construction of an in vitro model of CIRI in PC12 cells and analysis of Bmal1 gene and protein expression

**Figure 1.**
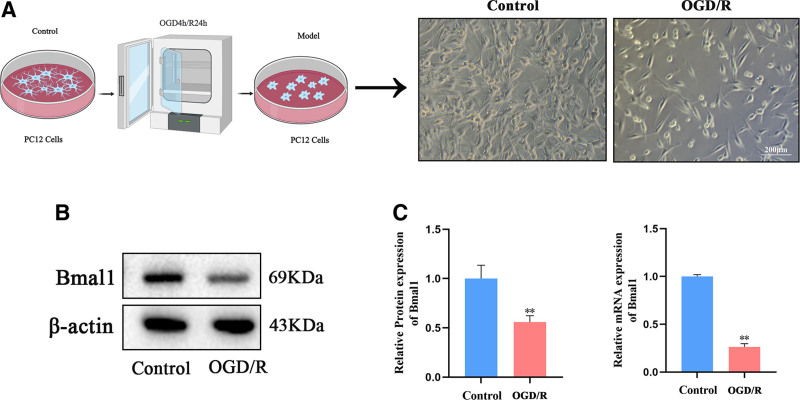
In vitro modeling of CIRI in PC12 cells, along with the expression of the Bmal1 gene and protein. (A) Schematic diagram illustrating the modeling of PC12 cells and their morphological changes observed under an inverted microscope before and after the procedure. (B) Western blot analysis was conducted to assess the expression of the Bmal1 protein. (C) RT-qPCR was performed to quantify the expression levels of Bmal1 mRNA. Data were presented as means ± standard deviations (n = 3). *P<.05 or **P<.01 compared to the control group. Bmal1 = brain and muscle Arnt-like 1, CIRI = cerebral ischemia-reperfusion injury, PC12 = pheochromocytoma cells, RT-qPCR = quantitative reverse transcription polymerase chain reaction.

To investigate the expression of the Bmal1 gene in CIRI, we observed cell crumpling, incomplete cell membranes, and an increase in dead cells in PC12 cells following OGD/R compared to the control group, thereby establishing the in vitro model of CIRI (Fig. 1A). In this in vitro model of CIRI, the expression levels of the Bmal1 gene and protein were reduced (Fig. 1B, C), indicating that Bmal1 gene expression was suppressed during the injury.

### 3.2. Construction of a stable overexpression model for the Bmal1 gene in PC12 cells and the establishment of a NC group

**Figure 2.**
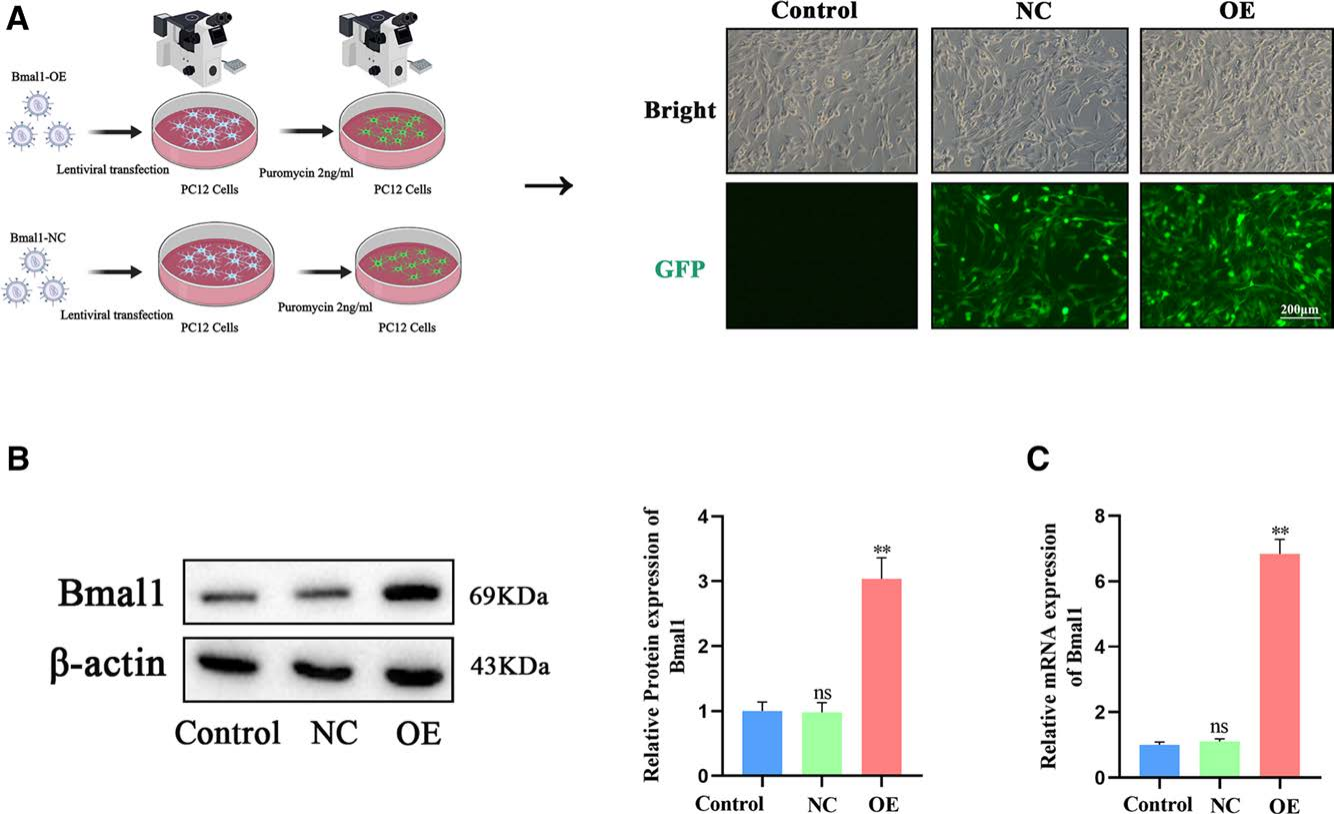
Stable overexpression of the Bmal1 gene in PC12 cells and construction of the negative control (NC) group. (A) Fluorescent expression results from the lentiviral transfection of the circadian clock gene Bmal1. (B) Western blot analysis was conducted to detect Bmal1 protein expression following lentiviral transfection. (C) RT-qPCR was performed to quantify Bmal1 gene expression following lentiviral transfection. Data were presented as means ± standard deviations (n = 3). **P < .01 compared to the control group, ns *P* > .05. Bmal1 = brain and muscle Arnt-like 1, NC = negative control, PC12 = pheochromocytoma cells, RT-qPCR = quantitative reverse transcription polymerase chain reaction.

The results indicated that green fluorescent protein expression was observed in the Bmal1 OE and NC groups under fluorescence microscopy, whereas no fluorescence was detected in the control group, confirming a transfection efficiency exceeding 95% (Fig. 2A). The mRNA and protein expression levels of the circadian clock gene Bmal1 were significantly higher in the Bmal1-OE group compared to the NC group (Fig. 2B, C). No statistically significant difference was observed between control and NC groups.

### 3.3. Effects of Bmal1 gene overexpression on cellular inflammation and apoptosis following OGD/R in PC12 cells

**Figure 3.**
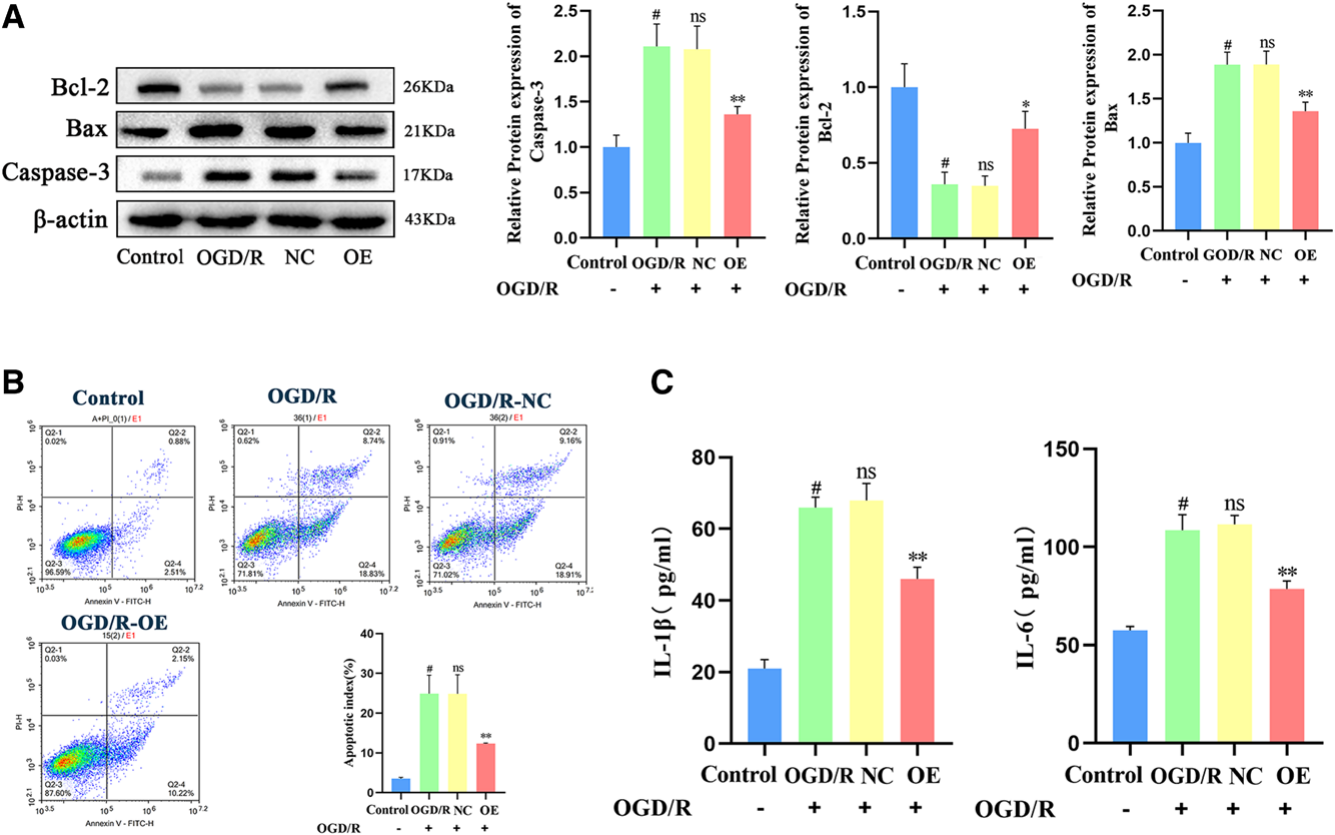
Effect of overexpression of the circadian clock gene Bmal1 on cellular inflammation and apoptosis following OGD/R in PC12 cells. (A) Western blot analysis was performed to detect the proteins Caspase-3, Bax, and Bcl-2. (B) Flow cytometry was conducted to analyze apoptosis. (C) ELISA was performed to quantify the inflammatory factors IL-1β and IL-6. Data were presented as means ± standard deviations (n = 3). #*P* < .01 compared to the control group. **P < .01 compared to the OGD/R group, ns *P* > .05. Bax = BCL2-Associated X, Bcl-2 = B-cell lymphoma-2, Bmal1 = brain and muscle Arnt-like 1, Caspase-3 = cysteine-dependent aspartate-directed protease 3, IL-1β = interleukin-1β, IL-6 = interleukin-6, OGD/R = oxygen-glucose deprivation/reoxygenation, PC12 = pheochromocytoma cells.

We employed Western blotting and flow cytometry to assess apoptosis and the expression levels of the apoptosis-related proteins Caspase-3, Bax, and B-cell lymphoma-2 (Bcl-2). The results indicated that, compared to the model group, the expression of pro-apoptotic proteins Caspase-3 and Bax significantly decreased, while the expression of the antiapoptotic protein Bcl-2 increased (Fig. 3A) and the apoptosis rate decreased (Fig. 3B). No significant differences were observed in the apoptosis rate or the expression of apoptosis-related Caspase-3, Bax, and Bcl-2 in the NC group. Subsequently, the effects of Bmal1 gene overexpression on the inflammatory IL-1β and IL-6 in PC12 cells following OGD/R were measured using ELISA. The results demonstrated that, compared to the model group, the levels of inflammatory factors IL-1β and IL-6 were significantly reduced in the Bmal1 overexpression group, while no significant differences were observed in the NC group (Fig. 3C). These findings suggest that Bmal1 gene overexpression can inhibit inflammatory factors in PC12 cells under conditions of oxygen-glucose deprivation and reoxygenation. Additionally, it can suppress the expression of apoptosis-related proteins and enhance the expression of antiapoptotic proteins, thereby alleviating apoptotic injury.

### 3.4. Effects of Bmal1 gene overexpression on oxidative stress markers in PC12 cells following OGD/R

**Figure 4.**
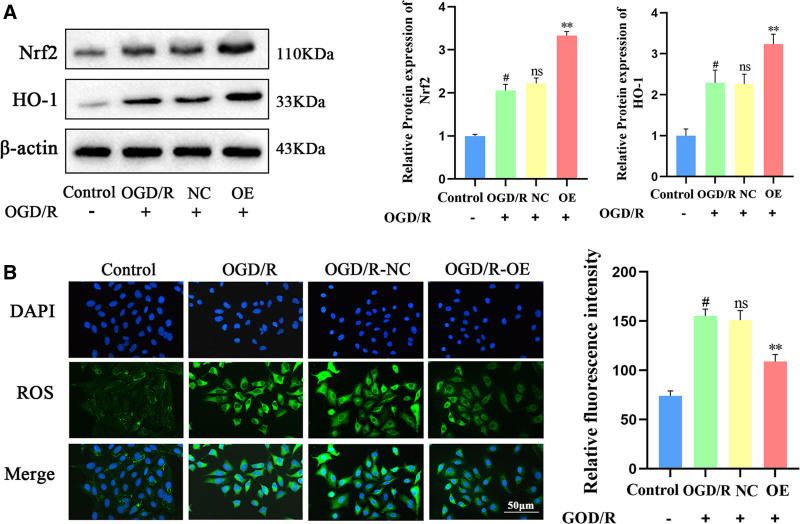
Effect of Bmal1 gene overexpression on oxidative stress markers following OGD/R in PC12 cells. (A) Detection of Nrf2/HO-1 pathway proteins by Western blot analysis. (B) Detection of ROS in PC12 cells using a fluorescent probe. Data were presented as means ± standard deviations (n = 3). #*P* < .01 compared to the control group. **P < .01 compared to the OGD/R group, ns *P* > .05. HO-1 = heme oxygenase-1, Nrf2 = nuclear factor erythroid 2-related factor 2, OGD/R = oxygen-glucose deprivation/reoxygenation, PC12 = pheochromocytoma cells, ROS = reactive oxygen species.

Previous studies have demonstrated that overexpression of the Bmal1 gene inhibits the inflammatory response and reduces apoptotic injury in PC12 cells subjected to OGD/R. CIRI is widely recognized to be associated with significant oxidative stress, leading to cellular inflammation and apoptotic injury. Thus, it is important to investigate whether the Bmal1 gene influences these processes by modulating the oxidative stress response. Consequently, we focused on the classical oxidative stress pathway, Nrf2/HO-1. We employed Western blot analysis to assess the impact of Bmal1 gene overexpression on the Nrf2/HO-1 pathway. The results indicated that Bmal1 gene overexpression significantly increased protein expression in the Nrf2/HO-1 pathway compared to the model group. In contrast, no significant difference in Nrf2/HO-1 protein expression was observed between the NC group and the model group. No difference in Nrf2/HO-1 protein expression was observed in the NC group compared to the model group. This suggests that the Nrf2/HO-1 pathway was activated following CIRI, and Bmal1 overexpression further up-regulated Nrf2/HO-1 pathway protein levels (Fig. 4A). The extensive release of ROS during ischemia/reperfusion induces lipid peroxidation, compromising cell membrane integrity and resulting in cell damage and death. We further examined the impact of Bmal1 gene overexpression on ROS levels in PC12 cells, a model for CIRI. Experimental results demonstrated that Bmal1 gene overexpression significantly increased the activity of superoxide dismutase (SOD) antioxidant enzymes while decreasing ROS release and malondialdehyde (MDA) production in PC12 cells (Fig. 4B, C). These findings suggest that Bmal1 gene overexpression effectively activates intracellular antioxidant proteins and enzymes, enhancing cellular antioxidant capacity. Consequently, oxidative stress damage following CIRI was mitigated, potentially reducing cellular inflammation and apoptotic cell death.

## 4. Discussion

CIRI is a common phenomenon in patients suffering from ischemic stroke. Oxidative stress is a key pathological aspect of CIRI, while inflammation and apoptosis are also significant mechanisms contributing to this condition. Excessive ROS following cerebral ischemia/reperfusion not only directly cause cell injury but also activate numerous inflammatory cytokines, including IL-6 and IL-1β, which are released rapidly. The rapid release of IL-6 and IL-1β exacerbates cell damage and death, while the activation of various apoptosis-related signaling pathways promotes neuronal apoptosis, leading to severe impairment of brain function.^[20–23]^ Bmal1 is the core gene of the circadian clock, and the Bmal1-Clock complex is a key regulatory element whose transcriptional activation and repression are driven by binding to the E-box on the per gene and is the primary basis for endogenous circadian rhythmicity in mammals.^[24,25]^ As a key regulatory element in the negative feedback loop of the clock gene, Bmal1 also regulates oxidative stress, inflammatory response, apoptosis, and autophagy in the body, thus regulating the onset, development, and prognosis of CIRI.^[26–28]^ As a core component in the circadian rhythm regulatory network, the Bmal1 gene has garnered increasing attention in neuroprotection research in recent years, particularly in the treatment of CIRI, where its potential regulatory mechanisms have become a research focus. Studies have found that the high-expression phase of the Bmal1 gene can significantly alleviate pathological changes caused by CIRI. For instance, in mouse models of cerebral ischemia-reperfusion, Bmal1 overexpression effectively reduces cerebral edema, diminishes infarct volume, and decreases neuronal apoptosis induced by ischemia. This suggests that Bmal1 overexpression may exert neuroprotective effects by regulating multiple biological processes, offering potential therapeutic value for improving brain function. Furthermore, studies reveal that in Bmal1-deficient cells, the regulation of oxidative stress and inflammatory responses becomes markedly dysregulated. Specifically, Bmal1 deficiency elevates intracellular ROS levels, induces hypoxia-inducible factor-1α accumulation, and suppresses the normal activity of the antioxidant factor Nrf2. These molecular alterations collectively drive excessive production of the pro-inflammatory cytokine IL-1β, potentially exacerbating inflammatory responses and tissue damage. This mechanism implies that Bmal1 plays a critical protective role in brain injury by modulating oxidative stress balance and inflammatory signaling. These findings demonstrate that the Bmal1 gene is not merely a circadian rhythm regulator but also exhibits complex and vital regulatory capabilities in inflammation, oxidative stress modulation, and neuroprotection. This discovery not only deepens our understanding of the molecular mechanisms underlying CIRI but also establishes novel strategies for intervening in brain tissue damage through targeted regulation of Bmal1 expression.^[29,30]^

Sirtuin 1 (SIRT1), a core member of the mammalian sirtuin protein family, plays a significant role in regulating oxidative stress and inflammation. At the transcriptional level, it exhibits circadian rhythms controlled by the Clock-Bmal1 complex. SIRT1 regulates circadian rhythms by activating Bmal1 transcription and is recognized as an enzymatic modulator of circadian rhythms. Studies demonstrate that the Sirt1-Bmal1 pathway also plays a crucial role in ischemic stroke, exerting regulatory effects in the early stages by modulating the expression of oxidative stress and inflammatory markers such as MDA, SOD, IL-6, and Tumor Necrosis Factor-alpha. Experimental evidence shows that melatonin enhances the expression of Sirt1-Bmal1 pathway-related proteins in a dose-dependent manner, significantly improving cell viability, antioxidant and antiapoptotic effects, and markedly enhancing autophagy, thereby protecting diabetic mice from CIRI.^[31,32]^ The Nrf2/HO-1 pathway is also one of the potential therapeutic targets for CIRI due to its significant antioxidant, anti-inflammatory, and antiapoptotic effects. Activation of the Nrf2/HO-1 pathway in models of CIRI has been shown to significantly reduce oxidative stress, inhibit inflammation and apoptosis, and exert a neuroprotective effect.^[33]^ Nrf2, a core transcription factor involved in the cellular response to oxidative stress, has been shown in several studies to activate the Bmal1 gene through the Nrf2 signaling pathway. The antioxidant and apoptotic responses it modulates protect cells from oxidative stress.^[34]^

**Figure 5.**
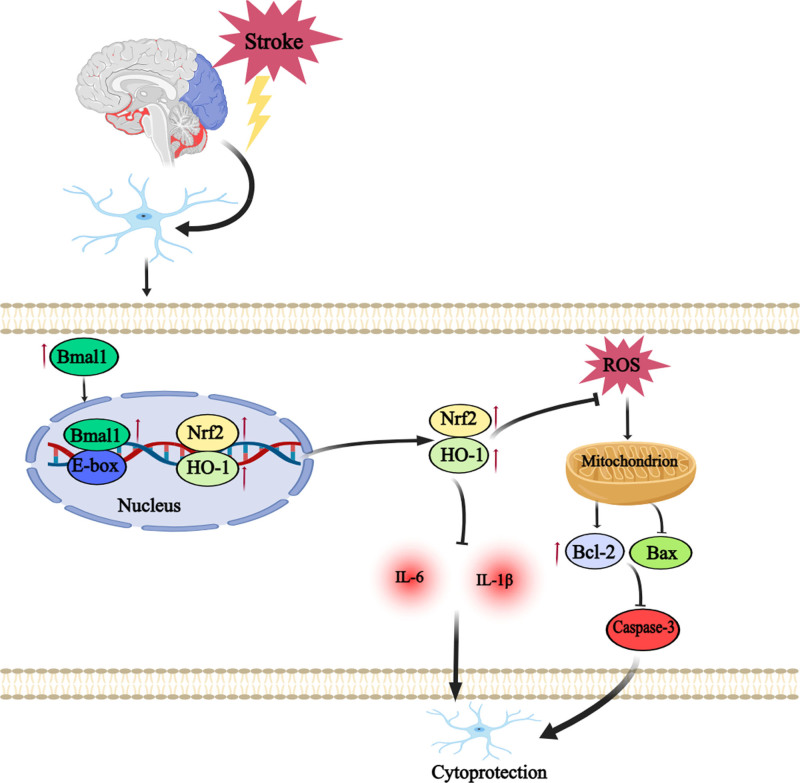
Mechanistic map illustrating the protective role of the Bmal1 gene in CIRI. CIRI = cerebral ischemia-reperfusion injury.

We aimed to verify whether the Bmal1 gene can reduce the cellular inflammatory response and apoptotic injury after CIRI by regulating the Nrf2/HO-1 oxidative stress pathway. To this end, we established a PC12 cell overexpression model of the Bmal1 gene through lentiviral transfection, alongside a NC model and an in vitro model of CIRI induced by OGD/R. Our results indicated that up-regulation of the Bmal1 gene significantly reduced the release of inflammatory cytokines IL-6 and IL-1β. Additionally, it inhibited the expression of pro-apoptotic proteins Caspase-3 and Bax while promoting the expression of the antiapoptotic protein Bcl-2, ultimately reducing cellular apoptosis. Further experiments revealed that up-regulation of the Bmal1 gene activated the Nrf2/HO-1 pathway, increasing the expression and activity of antioxidant enzymes such as SOD. This activation alleviated oxidative stress damage by scavenging excessive ROS and MDA, a lipid peroxidation end product. In summary, overexpression of the Bmal1 gene attenuates the cellular inflammatory response and apoptotic damage in CIRI, likely exerting a protective effect through regulation of the oxidative stress signaling pathway (Fig. 5). Although this study deeply explored the mechanism by which the Bmal1 gene regulates oxidative stress to attenuate inflammation and apoptosis in the OGD/R model using PC12 cells, there are several limitations. While PC12 cells are widely used in neuroscience research and offer many advantages, they are ultimately a cell line, which differs from in vivo neural cells and the environment of CIRI. Therefore, the results obtained from the PC12 cell model may not fully reflect the actual in vivo conditions of CIRI. Moreover, the specific role and mechanisms of the Bmal1 gene in an in vivo environment need further verification through animal models or clinical samples to enhance the applicability and generalizability of the study’s conclusions. Additionally, while the study defined the timing and conditions of OGD/R treatment, varying time points and treatment doses may significantly affect outcomes. Finally, although this study highlights the role of the Bmal1 gene, CIRI is a highly complex pathological process involving interactions among multiple genes and signaling pathways. Focusing solely on the Bmal1 gene may overlook other important molecules and mechanisms. We will continue to explore the role of the Bmal1 gene in CIRI, providing a new therapeutic perspective for future treatments.

## Author contributions

**Conceptualization:** Yuxing Zhang.

**Data curation:** MengJuan Wang.

**Funding acquisition:** Zhong Li.

**Investigation:** Zhong Li.

**Methodology:** Fukang Zeng.

**Software:** MengJuan Wang.

**Writing – original draft:** Fukang Zeng.

**Writing – review & editing:** Yuxing Zhang.
